# A Cryosectioning Technique for the Observation of Intracellular Structures and Immunocytochemistry of Tissues in Atomic Force Microscopy (AFM)

**DOI:** 10.1038/s41598-017-06942-1

**Published:** 2017-07-25

**Authors:** Eiji Usukura, Akihiro Narita, Akira Yagi, Nobuaki Sakai, Yoshitsugu Uekusa, Yuka Imaoka, Shuichi Ito, Jiro Usukura

**Affiliations:** 1 Structural Biology Research Centre, Graduate School of Science, Nagoya University, Nagoya, 464-8602 Japan; 2 OLYMPUS CORPORATION, Hachioji, Tokyo 192-8512 Japan

## Abstract

The use of cryosectioning facilitates the morphological analysis and immunocytochemistry of cells in tissues in atomic force microscopy (AFM). The cantilever can access all parts of a tissue sample in cryosections after the embedding medium (sucrose) has been replaced with phosphate-buffered saline (PBS), and this approach has enabled the production of a type of high-resolution image. The images resembled those obtained from freeze-etching replica electron microscopy (EM) rather than from thin-section EM. The AFM images showed disks stacked and enveloped by the cell membrane in rod photoreceptor outer segments (ROS) at EM resolution. In addition, ciliary necklaces on the surface of connecting cilium, three-dimensional architecture of synaptic ribbons, and the surface of the post-synaptic membrane facing the active site were revealed, which were not apparent using thin-section EM. AFM could depict the molecular binding of anti-opsin antibodies conjugated to a secondary fluorescent antibody bound to the disk membrane. The specific localization of the anti-opsin binding sites was verified through correlation with immunofluorescence signals in AFM combined with confocal fluorescence microscope. To prove reproducibility in other tissues besides retina, cryosectioning-AFM was also applied to elucidate molecular organization of sarcomere in a rabbit psoas muscle.

## Introduction

Atomic force microscopy (AFM) displays structures by tracing the surface of a sample with the sharp needle of a cantilever. The vertical movement following lateral scanning with the cantilever is not stable for long periods of time. Thus, the practical resolution is reduced compared to the theoretical potential resolution. Nevertheless, this technique has continued to be used in cell biology because AFM enables the observation of samples in liquid environments. Increases in scanning speed and stability and hardware improvements have gradually increased the practical resolution of the technique. Surprisingly, the movements of motor proteins and molecular shapes were captured using recent high-speed AFM techniques, although they were detected in a reconstituted system using purified molecules^1, 2^. AFM has not been widely used in cell biology, perhaps because unlike other microscopy techniques, intracellular structures are not usually observed directly in AFM. This limitation is because the cell membrane prevents access for the cantilever. Only the external surfaces of native or fixed cells had been observed using AFM until the development of the unroofing technique to prepare AFM samples. This technique was a breakthrough for the use of AFM because it enabled the direct visualization of part of the cytoskeleton at EM resolution in cultured cells^3–5^. However, the unroofing technique is effective only in cultured cells and does not work in tissues. For AFM to become a key type of microscopy in cell biology, it will need to achieve similar imaging capabilities as EM in terms of being able to display fine intracellular structures in tissues and having the capacity to be used to identify constituent proteins, which is similar to the process for immunohistochemistry. A cryosectioning technique was applied to solve this problem. To observe fine structures in tissues with AFM, several previous studies have used conventional plastic sections^6–12^. However, only the cell contours and some organelles were faintly detected because the cutting surface of the resin-embedded sample was flat without removing the resin. In EM, electrons that are scattered, absorbed and interfered by the samples form the image contrast. AFM forms images by touching and scanning the sample with a needle, but structures never appear unless the resin is removed. One study using plastic sections successfully embossed the inside structures by dissolving the surface of thin sections with alcohol^7^. Unfortunately, however, the contrast was not sufficient because there was insufficient embossing with alcohol. In fact, the complete removal of the resin without damaging the sample appears to be difficult after resin polymerization, even if the resin monomer is water-soluble. In many cases of choosing plastic sections, the specimens were fixed strongly with OsO_4_ and stained with lead citrate. The advantage of AFM, i.e., that it can be used in water and close to the native state, was wasted. Therefore, it is important to consider how the embedding medium is removed. This study focused on cryosections prepared using the Tokuyasu method^13–15^ in which samples are fixed slightly with glutaraldehyde and embedded in sucrose. Sucrose was expected to dissolve easily from the sample when soaked in buffer. The Tokuyasu method was originally developed for effective immunolabelling rather than structural analysis. Therefore, a capacity for immunocytochemistry in AFM would also be possible when using this method.

## Results

**Figure 1:**
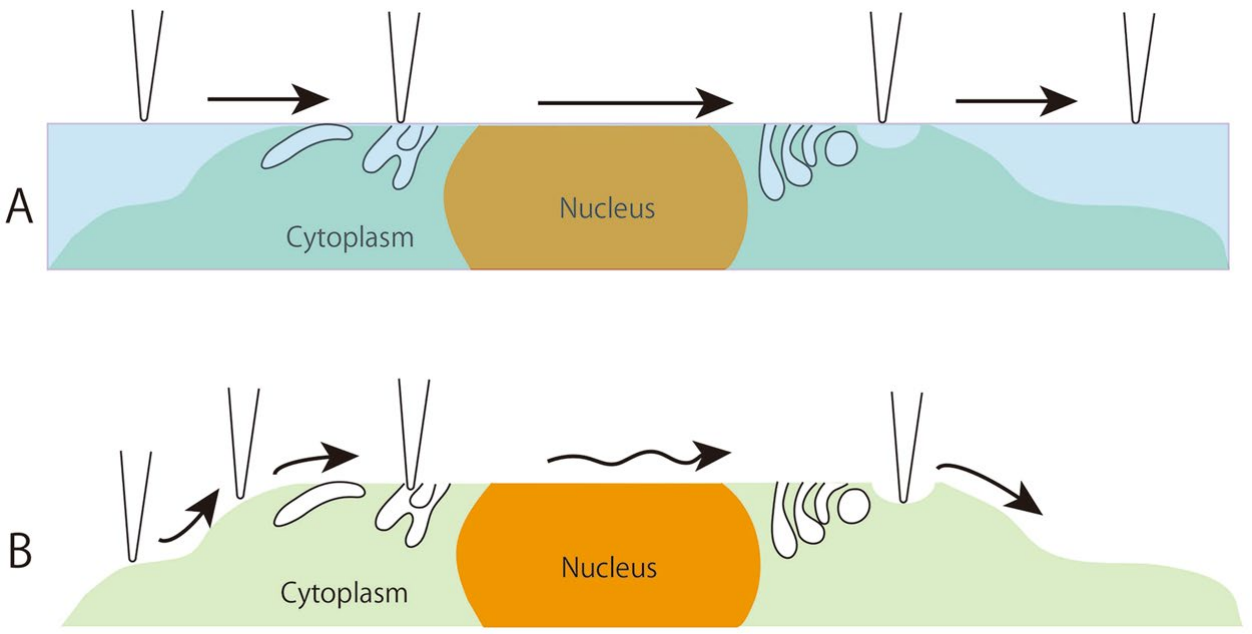
An illustration showing the difference in imaging between resin sections (**A**) and cryosections (**B**) with AFM. A: Polymerized resin is hard to remove; therefore, the cantilever just traces the flat surface of the resin section. B: For the cryosection, the embedding medium (sucrose) was removed by immersing the sections in PBS. The fine structures in cells and tissues were then exposed, which enabled the cantilever to trace along any undulations that appeared.

**Figure 2:**
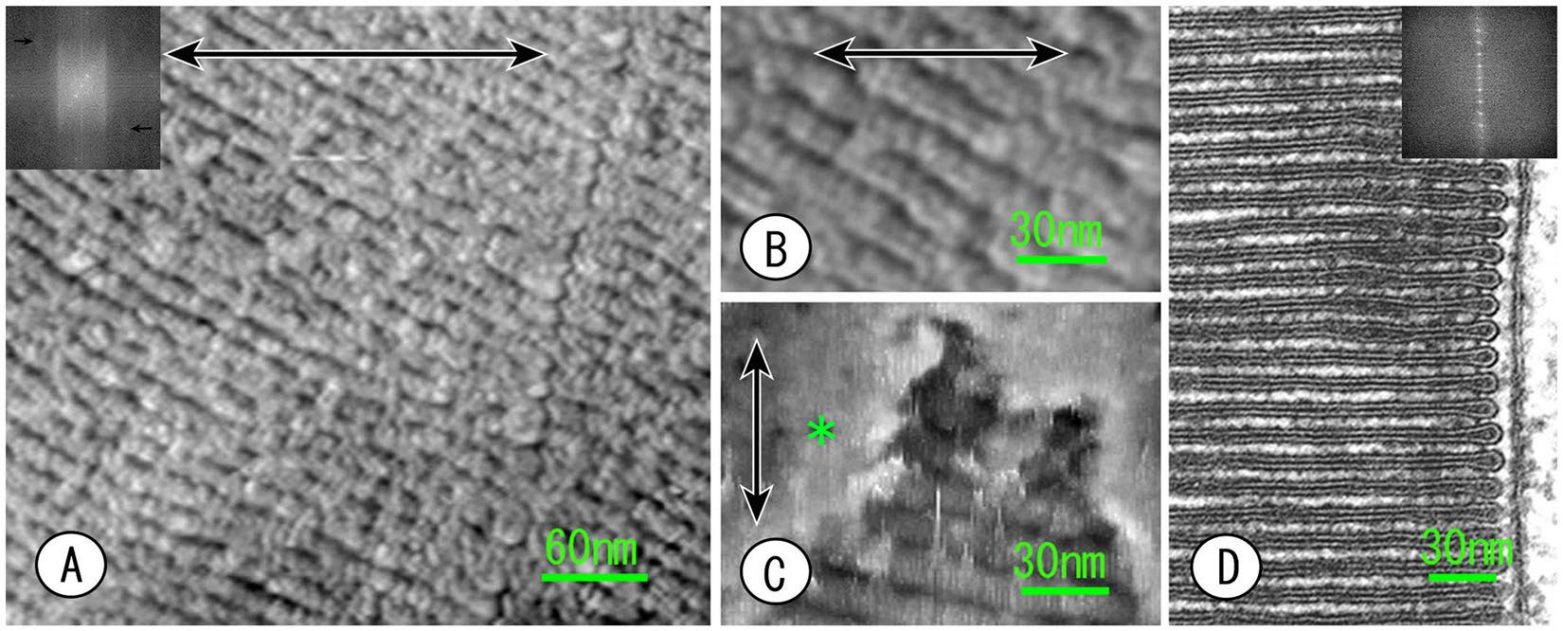
AFM images of the cryosections of photoreceptor rod outer segments after removing the sucrose embedding medium (**A**,**B**,**C**). Double-headed arrows indicate the scanning direction of the cantilever. (**A**) Highly ordered piling disks were observed, but the luminal spaces of the disks were almost collapsed, except at the incisures and the peripheral region (compared to the thin section EM image, **D**). (**B**) When enlarging the images of part of the disks, the luminal spaces look like slits. The unit membrane structure of the disks that appeared in D was not observed, but the structure appears to contain globular substances. (**C**) The marginal surfaces of the disk membranes are exposed but partially covered with cell membrane (asterisk) when the sections were near the peripheral region. (**D**) Thin-section EM image of the ROS inserted for a comparative analysis and better understanding of the ROS structure. The insets in A and D are Fourier transforms of A and B, respectively. The inset in A shows periodicity of disks piling up, but a high-resolution zone was obscured by another random structure, and there seems to be subtle information in the lateral direction of the disk membranes (arrows). Although the inset in D shows highly ordered periodicity of disks due to the contrast enhanced by the staining effect as though it were a line drawing, there is no information in the lateral direction of the disks.

**Figure 3:**
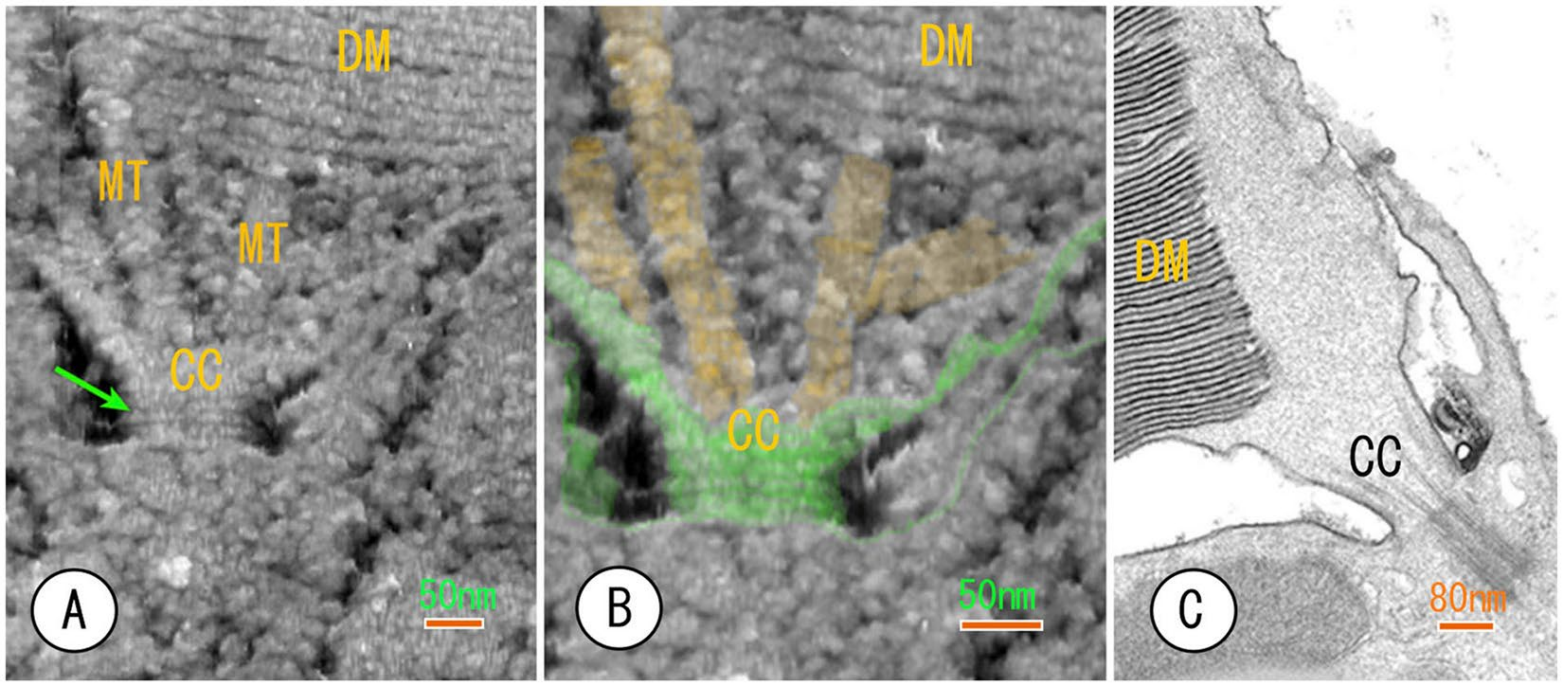
The AFM images of the connecting region between the outer and inner segment showing the outside and inside of the connecting cilium (CC). (**A**) The ciliary necklaces (an arrow) are exposed to the external surface of the connecting cilium because of the removal of sucrose. The upper section area contained microtubules (MT) that should be doubled in size. The finer structure of the microtubules is not observable. (**B**) The same image from A at a higher magnification. The external surfaces of the connecting cilium and microtubules are coloured in green and yellow, respectively. (**C**) A conventional thin-section EM image included as a reference.

**Figure 4:**
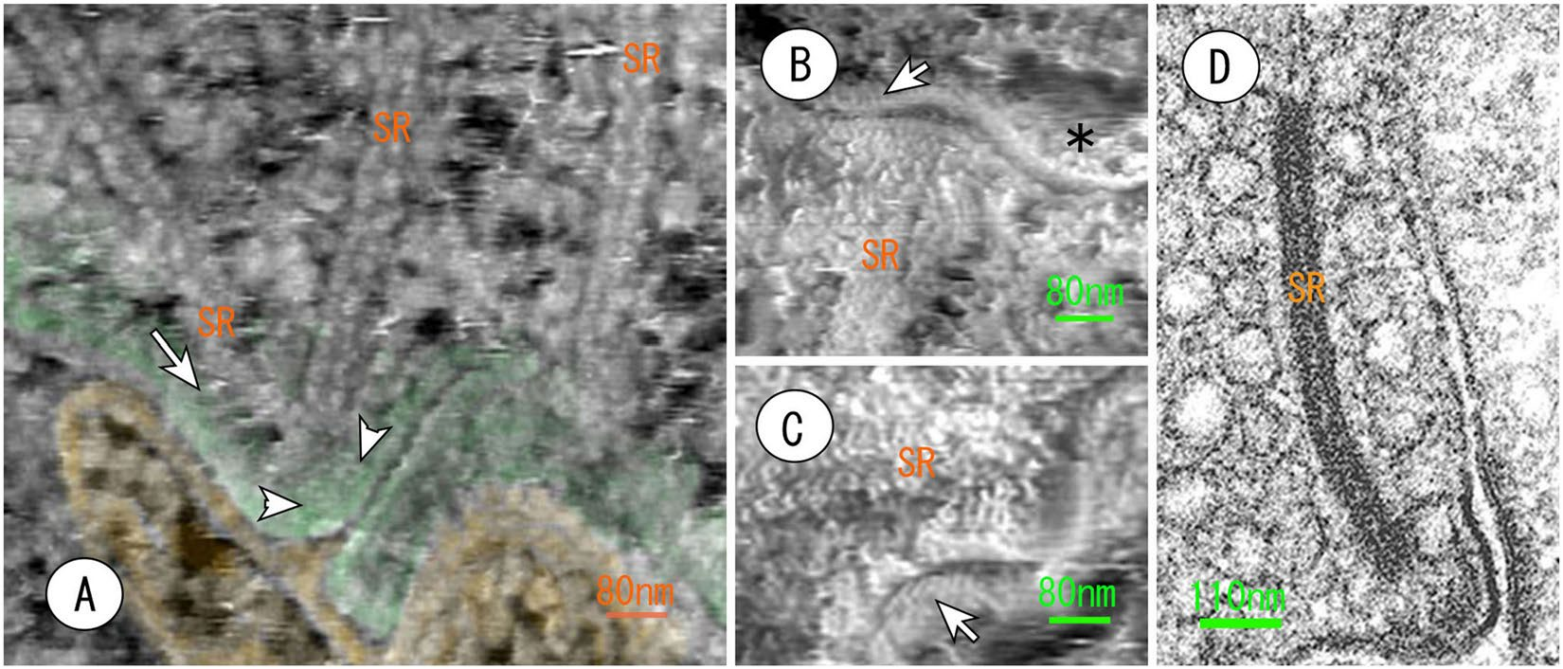
Atomic force micrographs of the synaptic active zone of photoreceptor cells (**A**,**B**,**C**). (**A**) The synaptic ribbons (SR) are tethered onto the cytoplasmic surface (green) of the pre-synaptic cell membrane with short filaments (arrow). The arrowheads indicate the characteristic circular structure on the cytoplasmic surface. (**B**) The post-synaptic membrane facing the pre-synaptic active zone contains several trans-membrane particles (arrow). An asterisk shows the cytoplasmic surface of the post-synaptic membrane. SR: synaptic ribbon. (**C**) The external surface of the post-synaptic membrane facing the active zone contains highly ordered particles (arrow). (**D**) A conventional thin section EM image in the synaptic active zone is provided in the inset for comparison. SR: synaptic ribbon.

Using the Tokuyasu method to prepare cryosections, the samples were fixed with 2% glutaraldehyde in the buffer and embedded in sucrose prior to freezing. Accordingly, the fine structures in tissues were well preserved throughout the preparation procedures, from freezing to sectioning and warming up. Whether the embedding medium can be removed easily after slicing is an essential point. As expected, sucrose could be dissolved and dispersed over time by immersing a section in phosphate-buffered saline (PBS). This removal enabled the cantilever to make direct contact with both the inside and outside surfaces of the cell membrane, organelles and cytoplasm in cells of tissues, as illustrated in Fig. 1. Cryosections from 150 nm to 400 nm in thickness were used, but the thickness was not appropriate for use in EM. Rather, the thickness was better for AFM because the membrane surface was exposed after removing the sucrose. A three-dimensional structure of membrane surfaces was never observed in conventional thin-section EM. After removing the embedding media, even if the sections were thin, AFM provided unique structural information different from that obtained from conventional thin-section EM. Several findings concerning the desmosome, Golgi apparatus, endoplasmic reticulum, mitochondria and additional structures have been obtained from analysis of the constituent cells in the retina. However, the present study evaluated chiefly the fine structures of photoreceptor cells, especially in the outer segments, the connecting cilium, and the synaptic regions that we are interested in. In order to assess reproducibility and availability of this technique, AFM imaging of muscle cells prepared by cryosectioning were described. To aid interpretation and compare this approach to alternate techniques, electron micrographs of thin sections are provided as insets in Figs 2, 3 and 4.

### Rod outer segments (ROS) of photoreceptor cells

The overview of ROS that the disk membrane piled up is enveloped by a cell membrane resembling a thin-section EM image but was different in the observations made by AFM at various points in detail. As a whole, the disk membrane appeared to be more three-dimensional with AFM (Fig. 2A,B and C). In the cases of grazing sections, the external surface of the cell membrane enveloping the disks was exposed widely because the embedding medium (sucrose) was removed (Fig. 2C). Although the disk is a flat sacculus, the lumen of the disk was mostly collapsed, except at incisures and marginal regions. Generally, the cell and organelle membrane appears as a unit structure (a tri-lamellar structure containing dark, light and dark layers), similar to a line drawing in thin-section EM (Fig. 2D). Therefore, the situation of the luminal space is understood at a glance in thin-section EM, even if it is very narrow or tightly collapsed. It was considerably difficult to recognize that a disk was a flat sacculus with AFM, but the lumen was barely registered as a groove at high magnification (Fig. 1B). The unit structure was not observed, regardless of magnification. Visual pigments (opsin) that are major intrinsic membrane proteins of the disks span the full thickness of the lipid bilayer of the disk membrane. Therefore, the unit structure found in conventional thin-section EM may be a staining effect with osmium tetroxide and lead citrate. Rather, granularity was represented in a disk membrane at high magnification with AFM (Fig. 2B). The arrangement of disks in the outer segments was quantified by Fourier transform. The periodicity of disk piling (approximately 28 nm) was preserved well in thin-section EM compared to AFM. In thin-section EM, however, the Fourier pattern in the vertical direction (piling direction) was emphasized as though it were a line drawing. It is thought that the osmium fixation and lead citrate staining enhanced the contrast and, inevitably, that the vertical periodicity of the disks was emphasized. In contrast, the Fourier diffraction image of the AFM contained a substantial halo, which hid a vertical reflection of the high-resolution zone. However, diffraction halo in AFM means, to some degree, that many random structures were contained in the arrangement of the disk membrane. Under careful observation, subtle spots seem to be found at the high-resolution (approximately 6 nm) zone in a direction perpendicular to disk piling.

### Connecting region between the outer and inner segments

The vertebrate photoreceptor is a highly polarized cell that is divided into three regions: the outer segment, the inner segment and the synaptic region. The connecting cilium bridges the outer and inner segments, as shown in Fig. 3A,B,C. The surface of the connecting cilium has been never observed three-dimensionally when using thin-section EM (compare Fig. 3A,B with Fig. 3C). However, the cantilever could access this surface in the extracellular space after the sucrose was removed. Thus, AFM could visualize ciliary necklaces (ciliary membrane specialization)^16, 17^ on the surface and inside the connecting cilium (Fig. 3A,B). This is the first AFM observation using PBS. Microtubules extending to the ROS inside the connecting cilium were also detected. However, the proto-filaments were not discernible in the microtubules because the microtubules in this area formed doublets and seemed to be modified by many associated proteins. Small granular substances filled the cytoplasm of the connecting cilium. It is still unclear whether such granularity reflects the real appearance of soluble components in cytoplasm or whether micelles of protein and/or sucrose remained behind the fine structures. Therefore, additional washes using PBS may be required to enable the detection of finer microtubule structures.

### Synaptic region

The photoreceptor cells form a recessing type of compound synapse, unlike the types found in the central nervous system. Special systems for recruiting synaptic vesicles to the synaptic release site (or active zone), the so-called synaptic ribbons^18–20^, are found in the synaptic region of the photoreceptor cells. The AFM displayed these synaptic ribbons three-dimensionally and further depicted the cytoplasmic surface of the pre-synaptic membrane as well as the outer surface of the post-synaptic membrane (Fig. 4A,B,C). The synaptic ribbons were tethered onto the cytoplasmic surface of the membrane at the active zone with short filaments, and a bilayer of globular substances was observed that was approximately 4 nm in diameter (Fig. 4A). This result was consistent with the results of previous experiments of freeze-etching EM^21^. Circular arrangements of small particles were observed on the cytoplasmic surface of the pre-synaptic membrane in active zones (Fig. 4A). This finding suggests that the synaptic vesicles dock in a structure similar to the mark of a kiss. In addition, cryosection AFM revealed an array of highly ordered particles on the outer surface of the post-synaptic membrane (Fig. 4C). After careful observation, these particles seemed to be trans-membrane proteins, as shown in Fig. 4B and C.

### Immunocytochemistry in AFM

**Figure 5:**
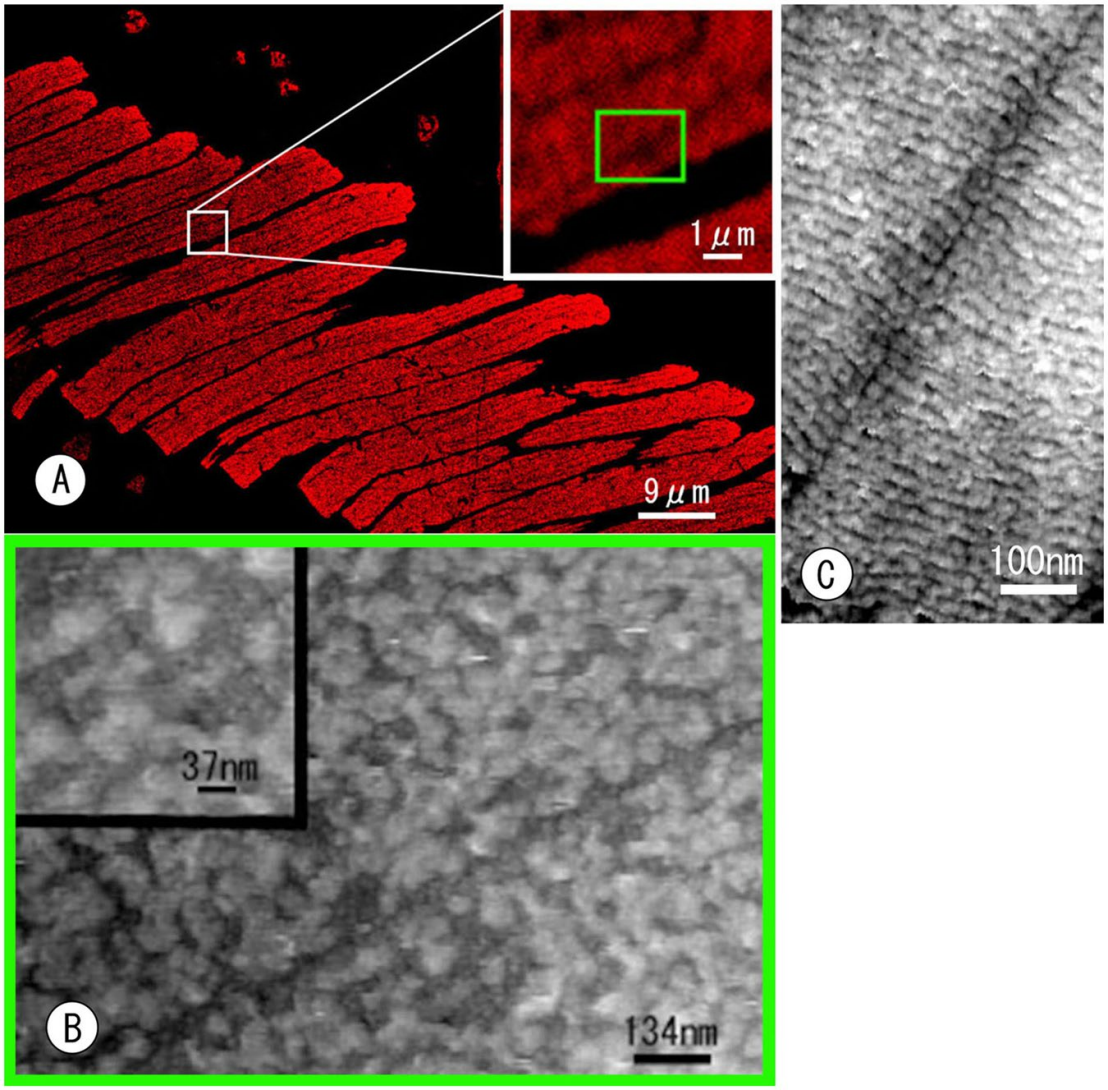
AFM immunocytochemistry identifying anti-opsin antibody binding (**A**,**B**). The anti-opsin antibody was used to evaluate whether AFM visualized the antibody binding because opsin is the major constituent protein that localizes to ROS. A: Immunofluorescence images of ROS labelled with the anti-opsin antibody and the secondary antibody conjugated to Alexa 568. The inset shows an enlargement of the white box. (**B**) Correlative AFM images of the green boxed area in A. Compared with the control (**C**), the anti-opsin antibodies conjugated with the secondary antibodies appear to have a complicated shape and bind heavily to disks in the ROS. The disk arrangement is also apparent behind the labelling at higher magnifications (inset). (**C**) Control AFM image incubated with non-immune IgG.

Currently, the AFM is a microscope measuring undulation of the specimen surface and/or force to receive data on the surface with a scanning needle. Therefore, AFM cannot distinguish whether detected particles are antibodies or other original binding proteins, even if a specimen is labelled specifically with an antibody. This is the first report on immunocytochemistry with AFM. ROS labelled with anti-opsin antibody was used in this study to make labelling easier to identify and because opsin (visual pigment) is a major intrinsic membrane protein. This is why the retina was chosen as the material for this study. To verify the immunolabelling in AFM, correlative analysis with confocal laser scanning microscopy (CLSM) was performed. An AFM (BIXAM) used in this experiment was combined with CLSM for simultaneous imaging through AFM and CLSM at a single location. The anti-opsin antibody was visualized using a secondary antibody conjugated with fluorescent dye (Alexa Fluor 568) for use with CLSM. Fluorescent signals for anti-opsin were specifically located at the ROS, and the ROS debris were shed and internalized into pigment epithelial cells (Fig. 5A). AFM successfully detected antibodies that were bound to the disk membrane along with fluorescent signals at the same time and location. CLSM detecting fluorescent signals emitted from the dye conjugated with a secondary antibody displayed the piling appearance of disks (Fig. 5A inset), even if the ROS was highly labelled. This occurred because fluorescence is emitted only from dye (small point in whole antibody). In contrast, in AFM images, piling disks in ROS were covered completely with irregularly shaped blobs while overlapping and fusing with each other (Fig. 5B), which is a feature of primary antibodies modified with secondary antibodies. Under careful observation at higher magnifications, however, the disks that were piled up were partially found behind the antibodies (Fig. 5B inset). Correlative observations with CLSM and control experiments consequently verified the specificity of the labelling. In principle, any antibody can be used in any tissue for this cryosectioning method. An evaluation of the AFM immunocytochemistry is discussed further below.

### Sarcomere of rabbit psoas muscle

**Figure 6:**
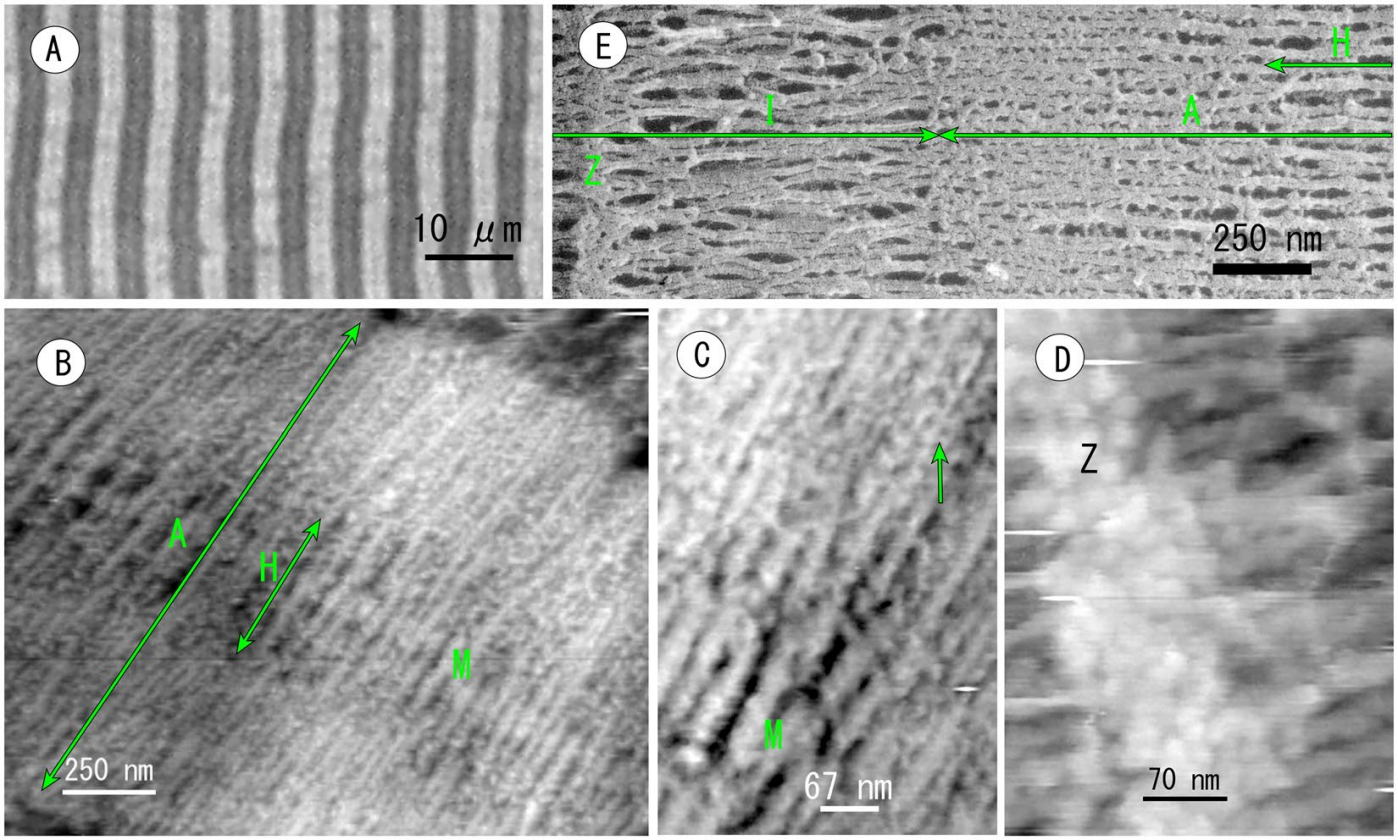
Atomic force micrograph of the cryosection of a rabbit psoas muscle. (**A**) Phase contrast light microscopic image of cryosections placed on a glass slide and soaked in PBS. Striations consisting of the anisotropic band and isotropic band were found. (**B**) AFM images of the anisotropic band in a sarcomere (a part of **A**) showing the interaction between thin (actin) and thick (myosin) filaments clearly. (**C**) High power AFM image of the anisotropic band showing thick filaments swelled in the centre of the H zone (M-line) and cross bridges (an arrow) between thin and thick filaments. (**D**) High magnification image of the Z-disk showing attachment of actin filaments. The main feature of the Z-disk remains ambiguous. (**E**) A freeze-etching EM image for reference. Half-length of a sarcomere is depicted. Z: Z-disk; I: isotropic band; A: anisotropic band.

Muscle fibres permeabilized with glycerination were observed by AFM after cryosectioning. This additional experiment was performed to prove reproducibility and the universal use of this preparation method in other tissues besides retina. As shown in Fig. 6, AFM displayed successfully the fine structure of the sarcomere at a resolution equivalent to EM. Cross bridges between thin (actin) and thick (myosin) filaments were recognizable in the anisotropic band (A band) (Fig. 6B,C). The myosin filament in the centre of the H zone (M-line) was modified by some blobs and was clearly thickened. Future experiments should resolve whether such blobs are represent to the M-line constituent protein. AFM also described the three-dimensional architecture of the Z-disk, but it remained ambiguous as to how actin filaments were organized in the Z-disk (Fig. 6D). Freeze-etching EM of the sarcomere is presented in the inset of Fig. 6E as a reference, which resembles the AFM images. Under careful observation of the high-power image of the Z-disk and M-line, and by considering that AFM measures structures in water without metal deposition, AFM images seem to include more structural information than offered by EM. In any case, AFM combined with this cryosectioning technique was useful for structural analysis of muscle cells and retinal tissue.

## Discussion

**Figure 7:**
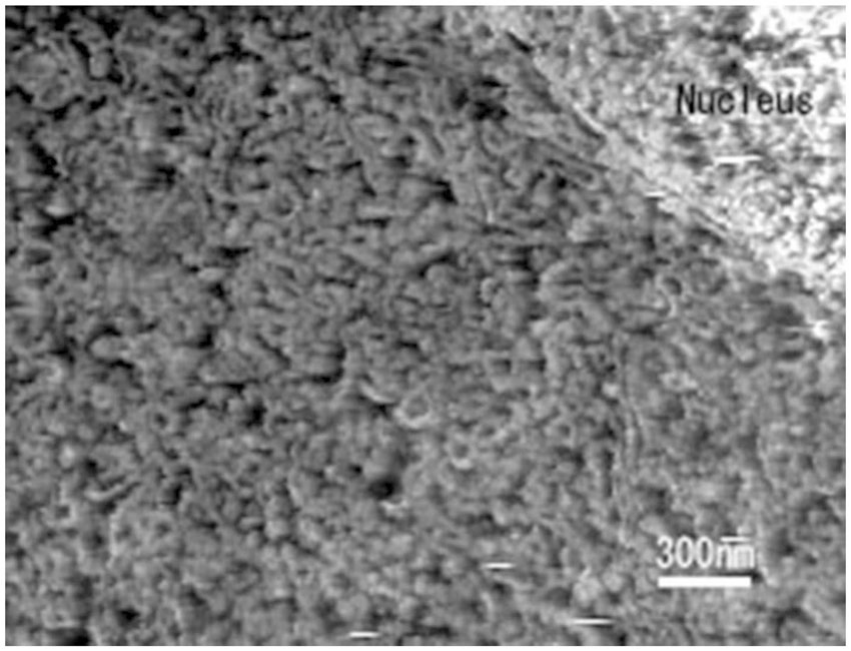
Atomic force imaging of the cryosections of a pigment epithelial cell in retina. The cytoplasm in the perinuclear region is occupied by a complicated network of membranous structures (likely smooth endoplasmic reticulum). A similar expanse of membranous structures has been not detected with thin-section or freeze-etching EM.

The structural information provided by AFM is different in principle from the information obtained by both EM and light microscopy (LM). Images that are obtained using LM and EM are formed by the scattering, interference and absorption of electrons or photons, but AFM produces an image by touching the surface of the specimen with a scanning needle. Therefore, AFM provides a different quality of structural information that is important for understanding fine structures. For example, the AFM of cryosections has revealed many tubular networks of membranous structures within the cytoplasm of pigment epithelial cells, as shown in Fig. 7, and these structures are mostly smooth endoplasmic reticulum (ER). In thin-section EM, these structures are observed as an assembly of small vesicles and are occasionally difficult to interpret because electron scattering is weak and similar to scattering from the embedding medium (resin). The network of the cytoplasmic ER has also been observed by using high-resolution CLSM^22^, although this technique could not discriminate rough ER from smooth ER. CLSM does not reveal the surface of the ER because the image is formed by fluorescence emitted from the dye labelling the membrane lipids.

The AFM images resembled freeze-etching EM images because both techniques provide the topography of the surface structure. In freeze-etching EM, ice in the tissue is sublimated in a vacuum to expose fine structures after the frozen samples are cut. Then platinum and carbon are evaporated to replicate the surface structure. Thus, the observation of a freeze-etching replica by transmission EM means visualizing the cutting surface. In our AFM technique, a cantilever scanning with a needle recorded the undulations of the surface of the cryosections after the embedding sucrose was removed. Removal of sucrose corresponds to etching (freeze dry). The major point of difference is whether the observation is performed in a vacuum with a transmission electron microscope after metal shadowing or in water with AFM without a metal deposit. Accordingly, the practical resolution of AFM seems to be higher than freeze-etching EM, as described in a previous study^5^, but the observation stability may be better for freeze-etching EM. Nevertheless, it is very important that AFM can be used to provide supplemental data for freeze-etching EM.

AFM has been exclusively used to observe the external surface of the cell or *in vitro* systems using purified proteins^23–27^. The use of cryosections in this study has enabled the use of AFM to depict fine intracellular structures in tissues, as opposed to the analysis of *in vitro* cultured cells. Several investigators have previously attempted to observe intracellular structures in tissue using AFM. However, suitable images were not acquired because these researchers used resin-embedded samples^6–12^. It is difficult to remove or dissolve the polymerized resin, even if the monomer is water-soluble. In a previous study, the surface of the resin section was treated with alcohol to emboss the fine structure^7^. The image becomes clearer than other AFM images of resin sections, but the image quality is not comparable to EM images. When embedding specimens into resin, specimens are usually fixed strongly with OsO_4_ to endure dehydration, heat-treatment and shrinkage in the polymerization process. Therefore, AFM observation using resin sections throws away an advantage of AFM in that AFM can be used to observe fine structures close to the cell’s native state in water. In contrast, in sucrose-embedded frozen sections, sucrose was removed easily from samples without using organic solvent. Unlike EM, observation in AFM does not depend on the thickness of the section. Rather, in thicker sections, the surfaces of the cell and the organelle membranes were detected widely and with three-dimensional properties. This is a finding that has not been obtained in thin-section EM. Additionally, immunolabelling was possible because the sample was fixed only slightly with 2% glutaraldehyde. The key goal of this study was the emphasis placed on sample preparation, which made it possible to remove the embedding medium (sucrose) used for cryosectioning. We hope that AFM will become a useful tool for histology and cell biology studies.

Therefore, important points of cryosection-AFM in cell biology are the universal use and reproducibility of this technique. To prove whether this method is useful in other tissues besides retina, cryosection-AFM was used to analyze the structure of muscle cells. In the present study, the M line and cross bridges between thin (actin) and thick (myosin) filaments were found in the anisotropic band (A band). The architecture of the Z line was also revealed at high resolution by cryosection-AFM. Accordingly, the cryosectioning technique offers sufficient reproducibility and universal use in histology and cell biology. This study excluded reproducibility arising from the skillfulness of the cryosectioning technique and the performance of the AFM hardware used. However, the cryosectioning technique should be straightforward for people who are familiar with microtome operation.

Another important aspect of this study is that immunocytochemistry is also possible for cryosectioned tissues and can be observed by using AFM combined with CLSM. AFM showed many irregularly shaped blobs covering ROS in cryosections labelled with the anti-opsin antibody. Such blobs were judged to be antibody binding only in AFM by comparing the images of control samples (compare Fig. 5B with Fig. 5C). In a strict sense, however, this is not conclusive evidence of the structure of an antibody, even if such blobs observed in labelled samples were not found in control samples. AFM cannot detect signals directly from the mark tagged to the antibody, such as fluorescence in LM immunocytochemistry or immuno-gold in EM. Therefore, the specific localization of antibody labelling cannot be evaluated with AFM only. Correlative analysis with CLSM and control experiments was necessary to verify the specific localization of the antibody. Several studies have tried to correlate materials with structures by comparing AFM images with the corresponding fluorescence images^28–34^. In this study, immunofluorescence was recorded together with the AFM images. Our AFM (named as BIXAM) is clearly correlated with CLSM.

In recent years, correlative EM and LM (CLEM) have become increasingly popular in cell biology, although the difference in resolution between EM and LM is too large to strictly correlate the difference between them. In other words, the fluorescence in LM shows the same distribution of labelled proteins in a given area compared to the distribution visualized when using EM. In such an area, numerous proteins exist that can be visualized using EM, and such target proteins cannot be identified unless they are double labelled with fluorescent dye and colloidal gold. In addition, computer-assisted matching is necessary to merge EM images with LM ones. In contrast, in AFM combined with CLSM, fluorescence and surface scanning imaging can be recorded simultaneously in water. Therefore, AFM images and correlative CLSM images can be merged exactly and more easily than they can in CLEM. A new type of correlative microscopy known as correlative atomic force and light microscopy (CALM) exists for this purpose. CALM is essential if immunocytochemistry is to be performed in AFM. Because AFM cannot detect the properties of materials, such as the signals from colloidal gold and fluorescent dyes, one cannot conclude that a structure displayed in an image is an antibody, despite the visualization of the molecule itself.

## Methods

The retinas used as samples in this study were isolated from 8 eyes of frogs (*Xenopus laevis*; supplied by a local animal dealer and another laboratory in Nagoya University) anesthetized by MS222 (Tricaine methanesulfonate, Wako Pure Chemical Industries, Ltd.; Osaka, Japan) (1 g/1 L), which is the most popular anaesthesia for fish and frogs.

Small parts of a rabbit psoas muscle were gifted from another laboratory, which was isolated under deep anesthetization just before experiments on actin purification were carried out in another laboratory. The provided muscle was immediately divided into several strips and tied to thin sticks in surgical sutures. The muscle strips attached to thin sticks were soaked in 50% glycerol in PBS to remove soluble components and stored for 2 days at −20 °C (so called glycerinated muscle).

All experiments were performed in according to Nagoya University regulations for the use of animals in research. The experimental protocol was proposed in advance and approved by the Institute of Laboratory Animals at Nagoya University (see the statement below).

The isolated 8 retinas were fixed with 2% glutaraldehyde in NaHCa buffer^35–37^ containing 30 mM HEPES, 100 mM NaCl, and 2 mM CaCl_2_ at pH 7.4, adjusted with NaOH, for 10 min and were then dissected into small pieces (approximately 1 mm^2^). The pieces were fixed for 2 hr with 2% glutaraldehyde in KHMgE buffer^35–37^ containing 30 mM HEPES, 70 mM KCl, 5 mM MgCl_2_, 3 mM EGTA at pH 7.4, which was adjusted with KOH. The fixed samples were washed three times (for 10 min each) with the same buffer and then moved to PBS without Ca (Ca free PBS).

Grycerinated muscles were warmed up to the room temperature, and washed three times (for 10 min each) with Ca free PBS, and then fixed with 2% glutaraldehyde in the same PBS. After fixation, samples were washed again three times (for 10 min each) with PBS to remove fixative.

The samples were prepared for cryosectioning according to the original Tokuyasu method^8–10^, except for the final steps. Fixed and washed samples were equilibrated with 2.3 M sucrose solution overnight. Subsequently, the samples were mounted on circular specimen carriers that are consumable parts of the Leica EM FC 7 cryomicrotome (Leica Microsystems, Vienna, Austria). The samples mounted on the carrier were quickly frozen by plunging them into liquid ethane cooled with liquid nitrogen.

Cryosections that were 200 nm to 300 nm thick were cut at −100 °C with a Leica EM FC 7 cryomicrotome and then transferred onto a glass slide using 2.3 M sucrose droplets on a perfect loop (Electron Microscopy Science, Hatfield, PA, USA). The glass slides used in this experiment were custom-made and consumable parts of the newest version of AFM (BIXAM; Olympus Corporation, Hachioji, Tokyo). These slides contain circular windows attached to thin glass covers surrounded by a frame printed using hydrophobic black ink (Matsunami Glass Industry, Ltd., Osaka, Japan). Cryosections placed on the circular window in this glass slide were warmed at room temperature and soaked in PBS immediately. To remove the sucrose completely, the sections were washed three times with PBS (for 10 min each) and left in PBS overnight. Finally, the samples were observed using AFM.

For immunocytochemistry, the sections were soaked in blocking solution containing 2% BSA (bovine serum albumin, fraction V) (Sigma-Aldrich; St. Louis, MO, USA) in PBS for 5 min after removing the sucrose. Then, the sections were washed once with fresh PBS. The sections were then incubated overnight with the primary antibody solution in a refrigerator. This study used the rabbit anti-opsin IgG raised against isolated bovine opsin. The anti-opsin antibody was diluted 1:250 with PBS containing 1% BSA and applied to the samples. For the negative control, samples were incubated with normal (non-immune) rabbit IgG (Santa Cruz Biotechnology, Inc., Dallas, TX, USA) diluted 1:200 in 1% BSA in PBS instead of the primary antibody, which was the anti-opsin antibody. Sections for both the labelling experiment and the control were washed six times with PBS (for 5 min per wash). Then the sections were incubated with goat anti-rabbit IgG conjugated to Alexa Fluor ® 568 (Invitrogen, Thermo Fisher Scientific, Inc.; Waltham, MA, USA) diluted 1:200 with PBS containing 1% BSA for 2 hr at room temperature. The sections were then washed six times with PBS (5 min each wash). The sections were observed with the new AFM technique combined with CLSM.

### AFM observations

The glass slide containing the sections soaked in PBS was mounted in a tip-scan type AFM (BIXAM)^38^, which was newly combined with CLSM (FV 1200; OLYMPUS CORPORATION; Hachioji, Tokyo, Japan). The cantilever precisely approached the target region of the retinal tissue as observations were made with CLSM. The images were recorded in the phase-modulation mode in which the amplitude domain was approximately 1–5 nm^39^. This AFM technique is characterized by a small and soft cantilever (2 μm wide, 9 μm long, and 0.1 μm thick in size) with a spring constant of 0.1 N/m (USC-F0.8-k0.1-T12-10: NanoWorld AG, Neuchatel, Switzerland). The radius of the scanning tip was approximately 8–10 nm. The resonance frequency of the probe tip was 0.8 MHz in the air and approximately 400 kHz in water. The maximum scanning range of the device was 4 μm (x axis) × 3 μm (y axis), and a range of 200–600 nm was scanned in this study. The scanning resolution was 320 pixels (x axis) × 240 lines (y axis).

### Thin section EM images inset in Figs 2, 3 and 4

These EM images are freeze-substitution thin sections. Frog retinas frozen rapidly were soaked in 1% OsO_4_ in absolute acetone cooled at −80 °C for two days^21^. Then, the samples were gradually warmed to room temperature and washed three times with fresh absolute acetone (5 min each). The samples were then embedded and sectioned in the conventional way. All sections were observed after staining with uranyl acetate and lead citrate.

### Freeze etching replica image inset in Fig. 6 for reference

Glycerinated muscles were heated to room temperature, washed three times (for 10 min each) with Ca-free PBS and fixed with 2% glutaraldehyde in PBS for 2 h. The fixed samples were washed again three times (for 10 min each) with PBS, assembled on the specimen holders and then frozen quickly by slamming the samples onto a copper block cooled with liquid helium. Frozen specimens were processed as described previously^40^.

### Animal experiments

E.U., A.N., J.U have licenses for experimental animal use in Nagoya University, which were issued after the authors received training on guidelines and regulations for animal use in research. Experimental protocols must be proposed in advance to the Institute of Laboratory Animals in Nagoya University. Experiments were performed with approval of the protocol in this study.
